# Optimizing the synthesis of CdS/ZnS core/shell semiconductor nanocrystals for bioimaging applications

**DOI:** 10.3762/bjnano.5.105

**Published:** 2014-06-27

**Authors:** Li-wei Liu, Si-yi Hu, Ying Pan, Jia-qi Zhang, Yue-shu Feng, Xi-he Zhang

**Affiliations:** 1 School of Science, Changchun University of Science and Technology, Changchun, Jilin, 130022, China; 2 International Joint Research Center for Nanophotonics and Biophotonics, Changchun, Jilin, 130022, China; 3 Department of gynaecology and obstetrics, China-Japan Union Hospital of Jilin University, Changchun, Jilin, 130033, China

**Keywords:** bioimaging, CdS/ZnS quantum dots, Pluronic F127

## Abstract

In this study, we report on CdS/ZnS nanocrystals as a luminescence probe for bioimaging applications. CdS nanocrystals capped with a ZnS shell had enhanced luminescence intensity, stronger stability and exhibited a longer lifetime compared to uncapped CdS. The CdS/ZnS nanocrystals were stabilized in Pluronic F127 block copolymer micelles, offering an optically and colloidally stable contrast agents for in vitro and in vivo imaging. Photostability test exhibited that the ZnS protective shell not only enhances the brightness of the QDs but also improves their stability in a biological environment. An in-vivo imaging study showed that F127-CdS/ZnS micelles had strong luminescence. These results suggest that these nanoparticles have significant advantages for bioimaging applications and may offer a new direction for the early detection of cancer in humans.

## Introduction

Over the past decade, the use of semiconductor nanocrystals or quantum dots (QDs) has developed remarkably due to their unique features. Compared to organic dyes, QDs have narrow emission peaks that can be systematically tuned from visible to near-infrared by manipulating their size, composition, and shape [1–7]. In addition, QDs exhibit a continuous absorption band behaviour that allows a single laser light source to excite multicolored QDs simultaneously. This is a major advantage compared with the simultaneous excitation of multiple organic dyes emitting at different wavelengths, which requires multiple light sources. All of these attractive features of QDs have made them extremely promising candidates for the new generation of optical probes for various immunoassays, multiplex imaging of cancer cells, and in vivo cancer targeting and imaging studies, etc. In 1998, Nie and Alivisatos were the first to report on the potential applications of QDs in biology [8–9]. There is no doubt that QDs offer a new tool for the multiplexed detection of target molecules and investigation into the intricacies of biomolecular interaction with cells [10–15]. With the development of synthesis QDs, an increasing number of QDs have been used for bioimaging and cancer detection applications.

Because the unique properties of QDs depend on their diameter, the control over the size of the ODs, their size distribution, crystallinity, and surface defects is crucial. Although they have good stability in terms of physics, the application of QDs in biology and cancer detection has been delayed due to their dispersion behaviour in aqueous solutions. It is critical to disperse QDs in water before their application by surface modification with biofunctional molecules for the purpose of bioimaging. However, for many biological applications, it is necessary to transfer the QDs into water through a capping ligand exchange [16–19]. There are many reports about the surface modification of QDs in the literature, for example, by functionalizing QDs with small molecules, e.g., sulfanylpropanoic acid, coating QDs with a silica shell, and encapsulating QDs within micelle polymer nanoparticles. Amongst the most-studied biocompatible polymer nanocarriers for in vivo studies there are Pluronic F127 triblock-copolymer micelle nanoparticles [20–21]. They can serve as a candidate for therapeutic and biomedical applications in vitro and in vivo.

A particular material for QDs, CdS, is an important direct-band semiconductor with a bandgap (*E*_g_) of 2.42 eV. By tailoring its composition and size, or surface functionality, it is possible to enhance luminescence emission and quantum yield of these QDs. The use of a coating agent can make CdS more stable. ZnS QDs are less cytotoxic than CdTe and CdSe QDs of the same size and surface functionalization, which demonstrates that the toxicity of cadmium QDs can be reduced by encapsulating them. Furthermore, it is possible to fabricate CdS/ZnS QDs that emit from the visible to the near-infrared (NIR) regions, and hence are suitable for bioimaging [22–23].

Several reports in the literature demonstrate the synthesis of CdS and CdS/ZnS QDs and their applications [24–27]. For example, Yongfen Chen et al. [28] reported the use of water-soluble luminescent CdS QDs as luminescence labels in cellular biology. Abdelhay Aboulaich et al. reported that the high photochemical stability of high luminescence-intensity CdS/ZnS core/shell QDs are suitable for optoelectronic devices and some biological applications [29–32]. Although CdS/ZnS QDs have been proposed for the use in biological applications, no research was so far reported on their biomedical applications. Our research has developed a novel integrative strategy that produces bioconjugated CdS/ZnS QDs for the use in bioimaging. In this study, we report a rapid and straightforward method for formulating CdS/ZnS QDs, encapsulated CdS/ZnS QDs in a hydrophobic core provided by block copolymer (Pluronic F127) micelles for bioimaging. The prepared nanoparticles were carefully characterized and their stability, toxicity, and optical properties assessed. Toxicity studies revealed that the micelle-encapsulated QDs do not exhibit toxicity within the timeframe at the cellular and tissue level. To the best of our knowledge, this is the first report using CdS/ZnS QDs as luminescence probes for bioimaging.

## Results and Discussion

### Characterization of QDs

**Figure 1:**
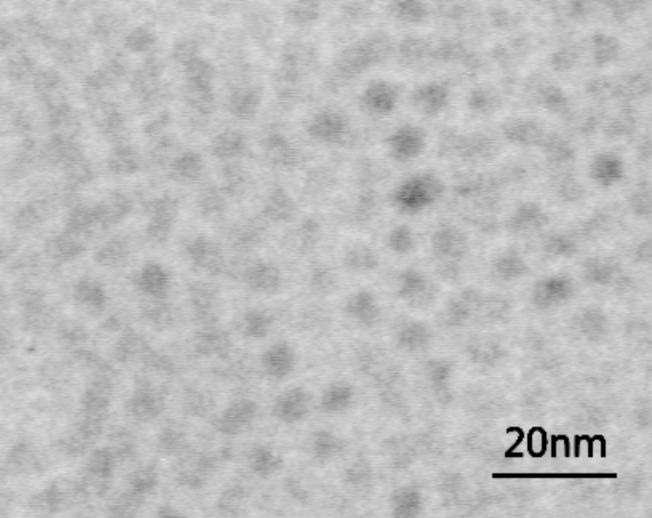
CdS/ZnS TEM.

Sizes and structure of the QDs were determined by using a JEOL JEM-100cx transmission electron microscope with an accelerating voltage of 80 kV. Transmission electron microscopy (TEM) images were taken by placing a drop of the particles in water onto a carbon film supported by a copper grid. Figure 1a shows the TEM images of organically dispersible CdS/ZnS QDs, from which their sizes are estimated to be 4.5 ± 1.7 nm.

**Figure 2:**
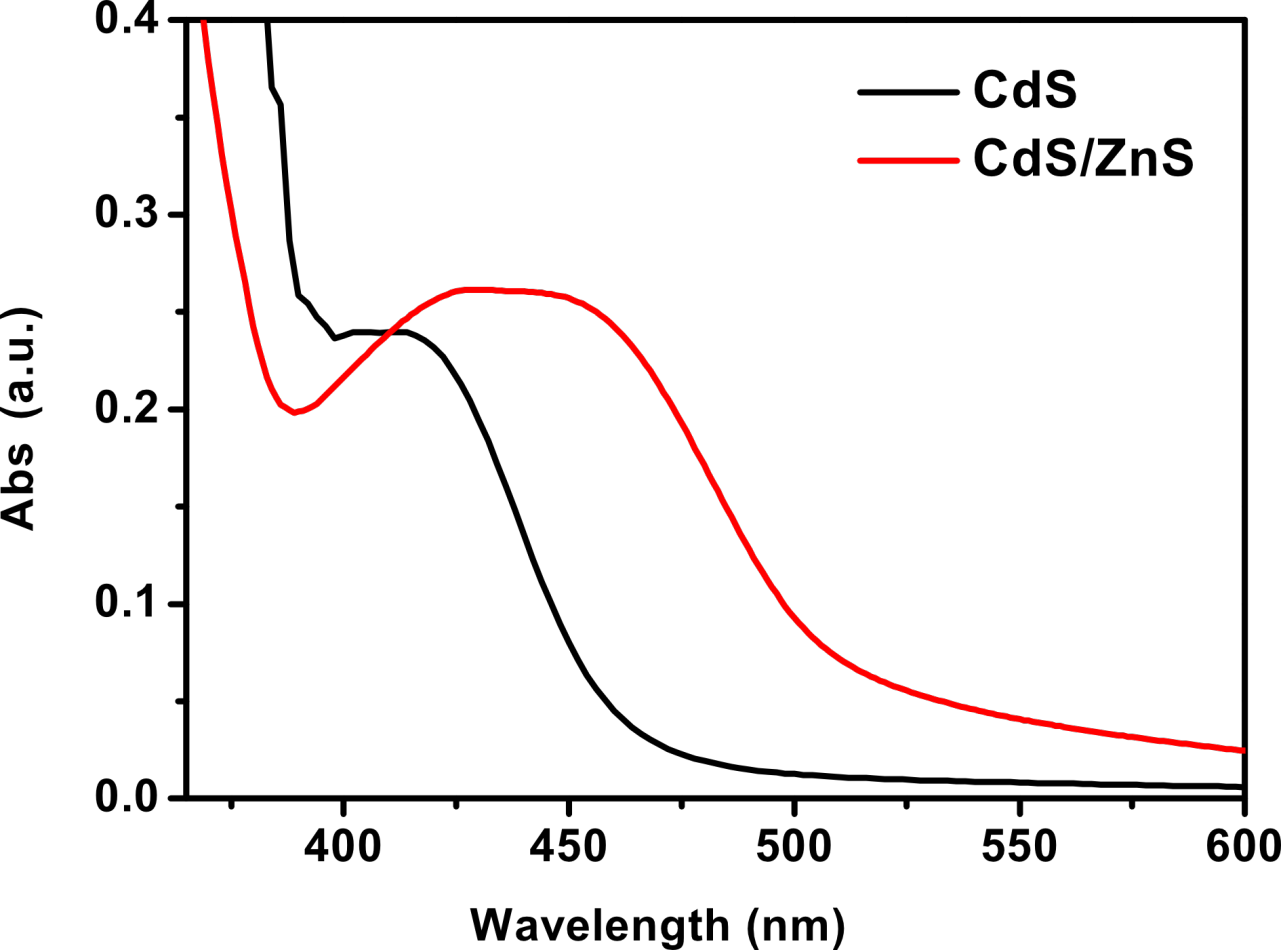
Absorption spectra for CdS and CdS/ZnS QDs.

The absorption spectra of QDs were collected by using a Shimadzu model 3101PC UV–vis–NIR scanning spectrophotometer over a wavelength range from 190 to 3200 nm. The samples were measured against chloroform as a reference. Figure 2 shows the absorption, all samples were used as prepared and loaded into a quartz cell from measurements. Comparing to CdS QDs there was a redshift of about 45–55 nm for CdS/ZnS QDs. This is due to the quantum confinement.

**Figure 3:**
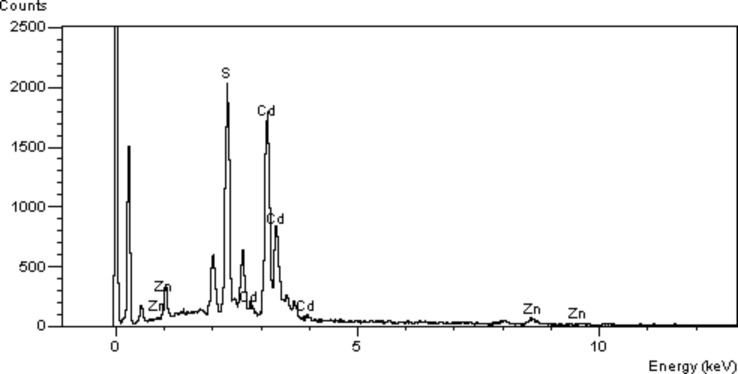
EDX spectra of the CdS/ZnS QDs.

CdS/ZnS QDs were further characterized by energy dispersive X-ray spectroscopy (EDX), as shown in Figure 3. The spectra exhibit the composition of the CdS/ZnS QDs and indicating Cd, S, and Zn as components of the CdS/ZnS QDs.

**Figure 4:**
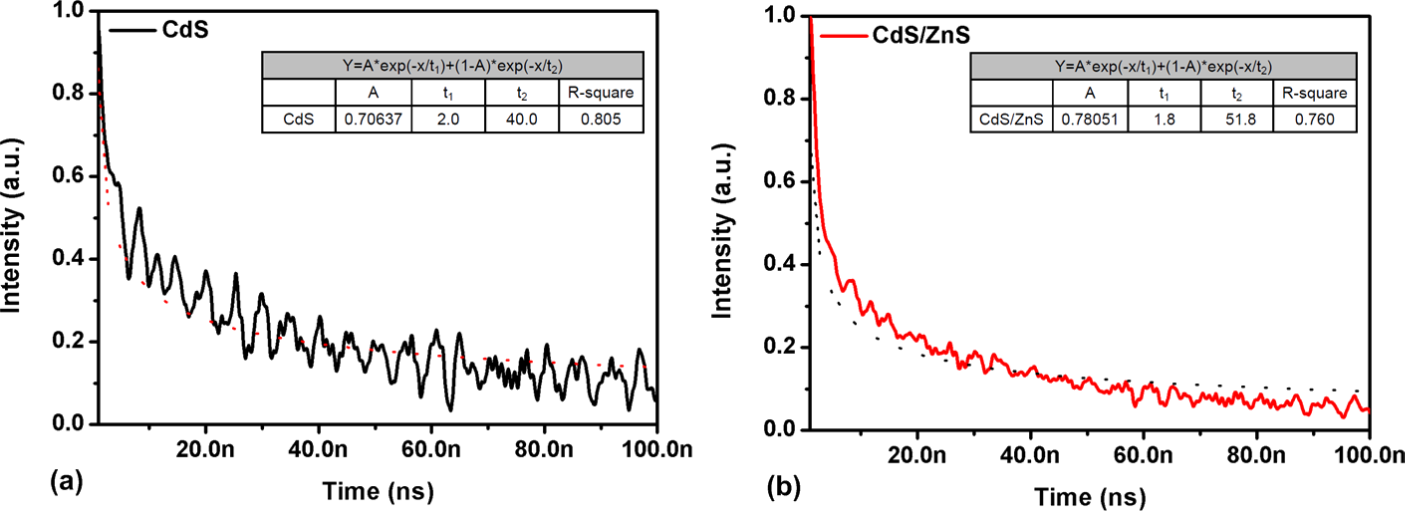
Fluorescence decay curves of (a) CdS and (b) CdS/ZnS QDs.

Photoluminescence (PL) decay is an important parameter for the study of the optical and surface chemical properties of nanoparticles. A multi-exponential decay curve provides evidence for the presence of nonradiative and radiative recombination. If the electron–hole pair recombines through the radiative pathway, a photon, which is associated with the bandgap of material, is emitted. Therefore in QDs with high QY, the non-radiative recombination is relatively weak. With regard to future optical studies, we performed a lifetime test. Figure 4 shows the measured normalized PL decay curves, measured by the double exponential curve (dashed line) with an EasyLife TCSPC system excited at 377 ns. The normalized decay curve can be well fitted by the double-exponential formula *y = A·*exp(−*t*/*t*_1_) *+* (1 − *A*)·exp(−*t*/*t*_2_)*.* Based on the lifetime data, the decay lifetime was estimated to be 50 ns for CdS/ZnS QDs, and 38 ns for CdS QDs. ZnS has a major influence on the lifetime decay profile of CdS/ZnS QDs. Trapping states are caused by surface defects located within the bandgap, which lead to the rise of nonradiative recombination.

### Emission spectra

**Figure 5:**
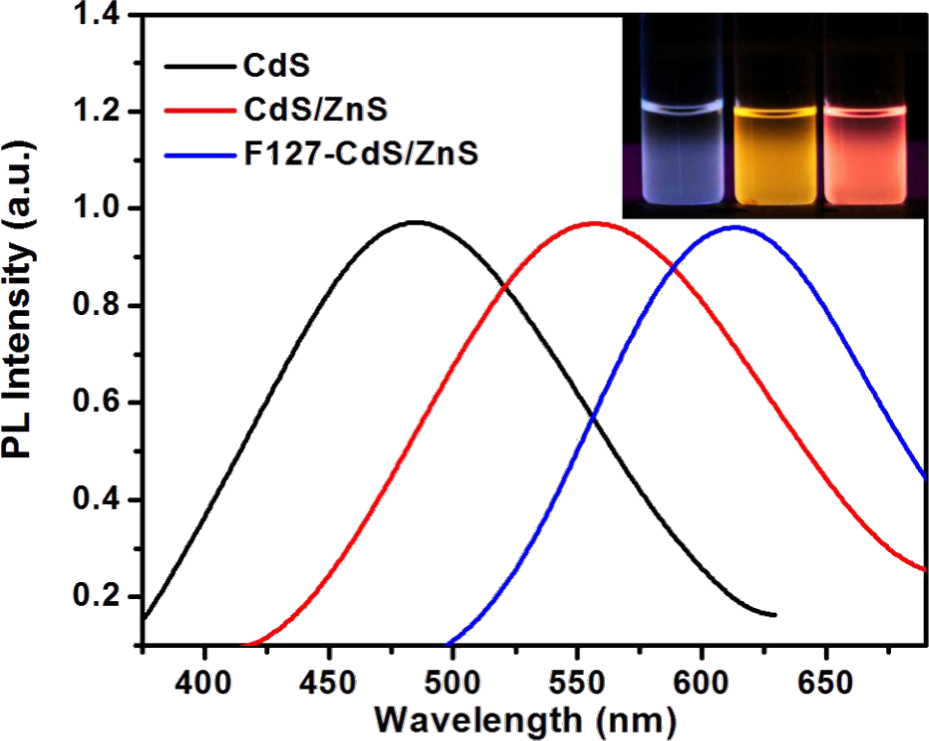
Emission spectrum for three samples.

The emission spectra were collected by using a Fluorolog-3 spectrofluorometer. The excitation wavelength was set at 440 nm and the emission was scanned from 480 to 900 nm. The result is shown in Figure 5 for CdS/ZnS and F127-CdS/ZnS QDs. Both of them exhibit a red shift compared with CdS QDs. The emission spectra are broad and the full width at half maximum is noted to be around 90 nm. This width was attributed to the wide size distribution and composite luminescence from the defect energy levels of the nanoparticles. Because the optical properties of QDs depend on the size, the absorption and emission characteristics of the QDs are closely related to the dendrimer template for preparing the QDs [33–35]. Because of the encapsulation, there is a change in the dielectric constant of the surrounding medium of the QDs, which can also be accounted for a shift of the emission wavelength.

Extensive research on the properties of QDs revealed that core/shell-structured QDs are desirable due to their strongly enhanced quantum yield (QY). We also tested the QY of three samples based on Equation 1:

[1]
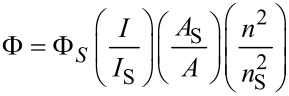


Here, Φ and Φ_S_ are the QYs for the sample and the standard, *I* (sample) and *I*_S_ (standard) are the integrated emission peak areas, *A* (sample) and *A*_S_ (standard) are the absorption values at the excitation wavelength, and *n* (sample) and *n*_S_ (standard) are the refractive indices of the solvent used, respectively. RG6 was used as reference. The QYs of CdS QDs, CdS/ZnS QDs and F127-CdS/ZnS reached up to 36%, 42%, and 65%, respectively. We can see that the core/shell structured QDs showed high QYs, outstanding physical and chemical stability, this strongly enhanced QY could be attributed to the wide band gap ZnS shell providing surface trap states that enhance the photostability.

### Photostability test

**Figure 6:**
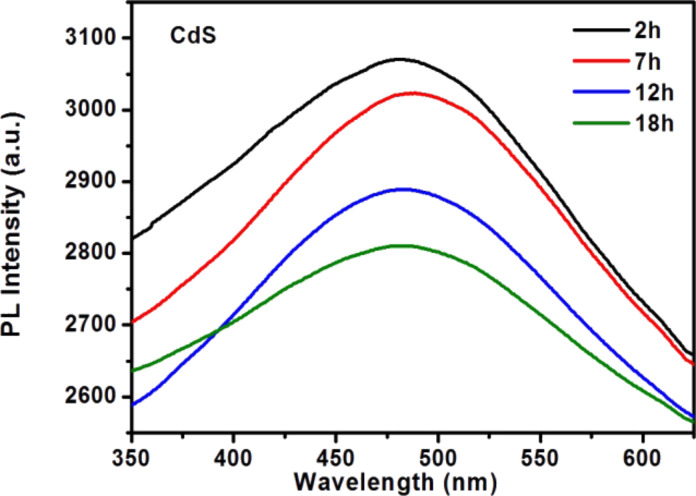
CdS PL spectra for photobleaching.

**Figure 7:**
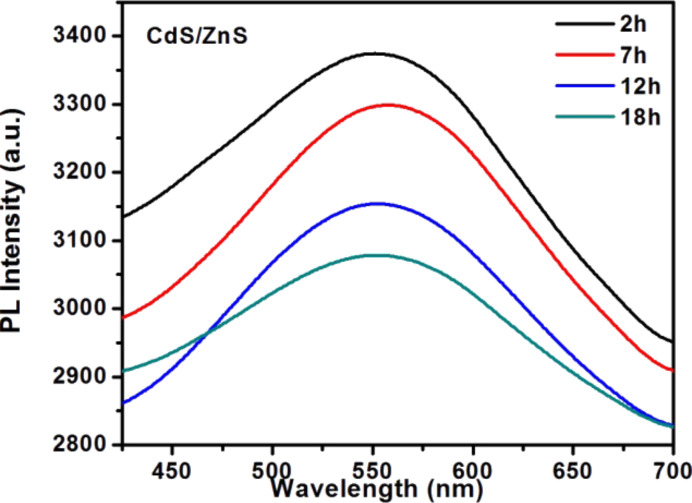
CdS/ZnS PL spectra for photobleaching.

**Figure 8:**
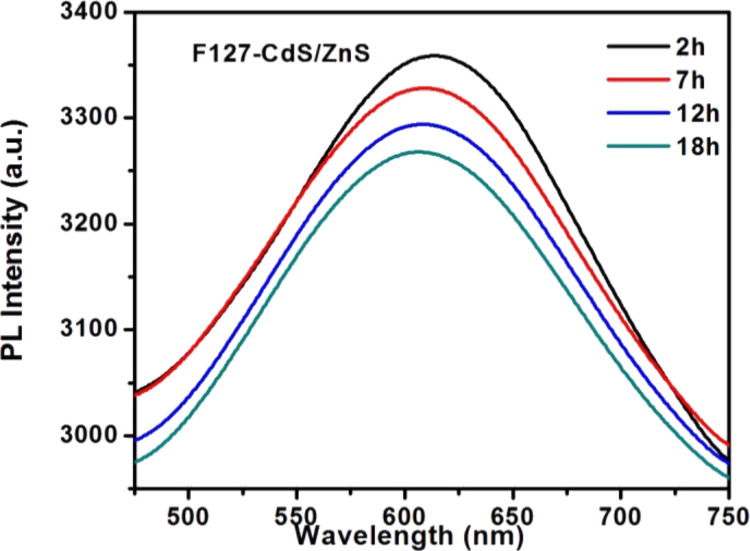
F127-CdS/ZnS PL spectra for photobleaching.

Photobleaching is an effective method for improving the bioimaging applications of nanocrystals especially for core/shell structured QDs. We carried out photostability experiments of F127-CdS/ZnS irradiated by continuous-wave (CW) laser with a wavelength of 447 nm and power density of 0.5 W/cm^2^. PL tests with different irradiate times were carried out. As Figure 6, Figure 7 and Figure 8 show, the results indicate that photobleaching has occurred. The experimental results revealed that F127-CdS/ZnS exhibits excellent photostability compared to the CdS QDs. CdS/ZnS QDs have almost the same photostability as F127-CdS/ZnS, and the PL intensity of F127-CdS/ZnS and CdS/ZnS QDs change less significantly after laser irradiation. This indicates that the bioconjugation with F127 did not affect the stability of the CdS/ZnS QDs. CdS and ZnS are group II–VI semiconductors with a similar crystalline structure and a lattice mismatch of 6.4%. As ZnS is a phosphor material with a high bandgap energy of 3.66 eV, the use of ZnS leads to enhanced stability and luminescence intensity. The ZnS protective shell not only enhances the brightness of the QDs but also improves their stability in a biological environment. This study provides a useful synthetic route for producing water-dispersible nanocrystals that should easily be applicable in biology.

### Effect of pH on the hydrodynamic diameter of the QDs

**Figure 9:**
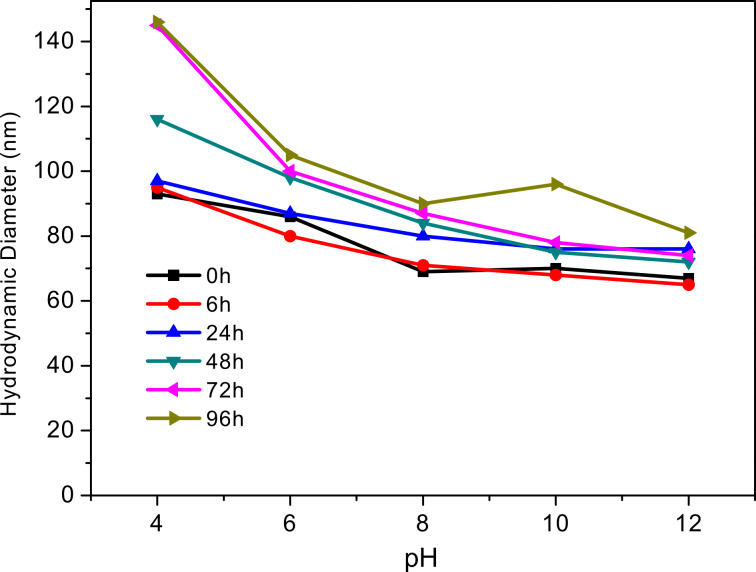
Hydrodynamic diameter measured by DLS for F127-CdS/ZnS QDs at different pH values.

The dynamic light scattering (DLS) technique was used to measure the hydrodynamic diameter profile of the micelle-encapsulated nanocrystals. For the purposes of the DLS analysis, the thickness of the adsorbed polymer layer was determined according to Equation 2 [36]:

[2]
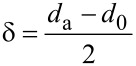


where δ is the width of the polymer layer, *d*_a_ is the hydrodynamic size after polymer adsorption and *d*_0_ is the hydrodynamic size of the bare nanospheres. However, the bare nanospheres were not stable. From this equation, we determined that the dispersion in water and the pH value can affect their stability. We used DLS as an accurate approach to monitor the colloidal stability of F127-CdS/ZnS micelles. The DLS samples were diluted in HPLC water (1:30). Then, we investigated the formulation of the micelle-encapsulated nanoparticles under various pH values, testing the stability of the F127-CdS/ZnS QDs. The variation of the hydrodynamic diameter under different pH values is shown in Figure 9. The result suggests that their stability is not affected by pH values.

### Cell imaging and viability studies

**Figure 10:**
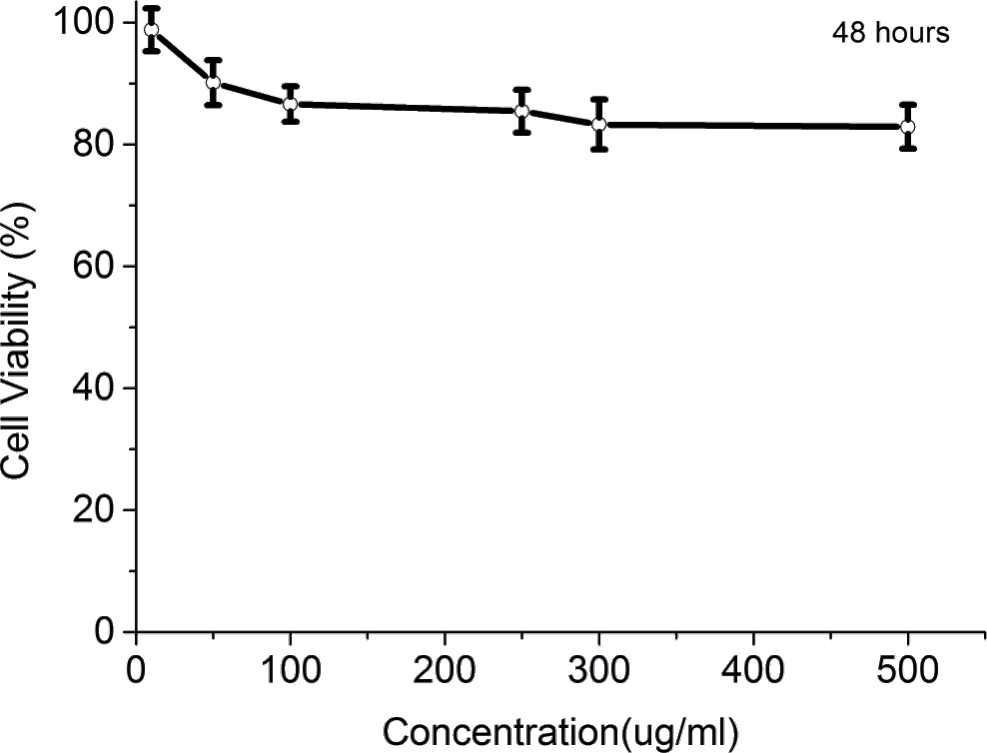
Viability of Panc-1 cells after 48 h of treatment in the presence of F127-CdS/ZnS QDs.

For in vitro studies, a cell viability (MTS) assay was carried out for F127-CdS/ZnS QDs. As shown in Figure 10, we tested the cell viability of Panc-1 cells, treating them with various concentrations of ternary nanocrystal formulations for 48 h. The cell viability remained at 84% even at a concentration as high as 500 µg/mL. This experiment demonstrates the minimal cytotoxicity associated with these nanoparticle formulations.

**Figure 11:**
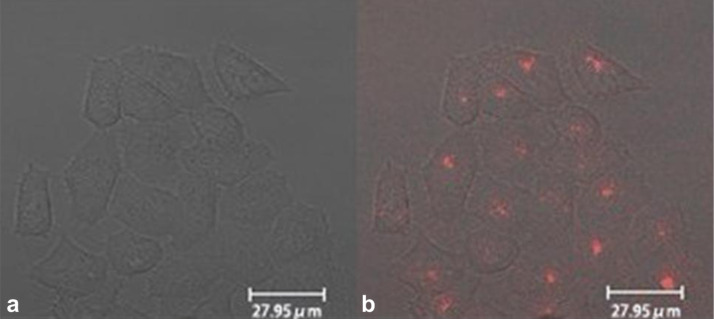
Confocal microscopic images of Panc-1cell treated with micelle encapsulated CdS /ZnS (a) transmission image, (b) overlay image.

For the bioimaging study, it is well known the cell membrane folate receptor is a potential molecular target for tumor-selective drug delivery, and the folate conjugates can be used to target pancreatic cancer. In our study, in vitro confocal microscopy studies were performed to re-confirm the uptake of F127-CdS/ZnS QDs bioconjugates in Panc-1 cells. Panc-1 cells were treated with F127-CdS/ZnS QDs for 2 h. Subsequently, the cells were rinsed with a PBS buffer to remove free nanocrystals and imaged by using confocal microscopy. Figure 11 shows robust cellular uptakes of the ternary nanocrystal samples. The red singnal is from F127-CdS/ZnS QDs, and background fluorescence and autofluorescence from the cells have been successfully suppressed. We can see that there is no damage to the cells, indicating the low toxicity of the formulated nanoparticles.

### Animal studies

**Figure 12:**
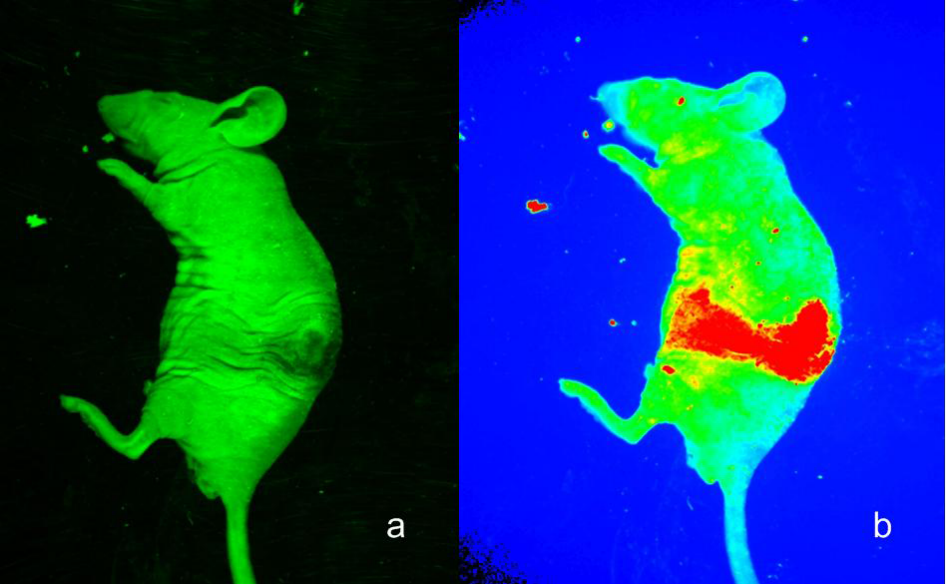
In vivo whole body imaging of nude mouse injected with F127-CdS/ZnS QDs, (a) transmission image, (b) luminescence imaging.

Five-week-old nude female mice were purchased from the experimental animal center of Jilin University. The animal housing area was maintained at 24 °C with a 12 h light/dark cycle. The animals were fed ad libitum with water and standard laboratory chow. The F127-CdS/ZnS micelles were injected into the nude mice subcutaneously, and the imaging was retrieved with a Maestro optical system with spectrally optimized lens system and a 470 nm laser. Images were recorded with a scientific-grade megapixel CCD sensor. The result is shown in Figure 12. Figure 12a is the bright field image, and Figure 12b is the overlay image. We found that there was a bright emission where F127-CdS/ZnS micelles were injected. In addition, we determined that the signal of the QDs (red) can be separated from the autofluorescence background (green). This in vivo imaging indicated the great potential of 470 nm laser excitation for autofluorescence-free QDs-based animal imaging. Further studies involving a systemic administration of the nanoparticles via the intravenous route and their subsequent studies of the biodistribution are required to gather information about the in vivo toxicity and the excretion from the animal body.

## Conclusion

In summary, we have prepared nanoparticles as excellent nanoprobes for bioimaging applications. CdS/ZnS QDs encapsulated in biocompatible amphiphilic Pluronic F127 have a good photo-stability and were successfully used for in vitro and in vivo studies. In addition, from our study of cell viability, the prepared ternary nanocrystals were shown to have a low cytotoxicity. Given their biocompatibility and the high efficiency in relation to targeted delivery, these nanoparticles have great potential to serve as a new generation of multifunction agents for clinic diagnosis and treatment.

## Experimental

### Materials

Cadmium oxide (CdO, 99.9+%), oleic acid (OA, 99%), sulfur (S, 99%), zinc chloride anhydrous (ZnCl_2_, 98+%), sodium diethyldithiocarbamate trihydrate (Na(DDTC), 98%), were purchased from Alfa Aesar. 1-Octadecene (ODE, 90%), oleylamine (OAm, >50.0%), Pluronic F127 were purchased from Sigma-Aldrich. Ethanol (99.8%) and chloroform (99.0+%) were purchased from Acros Organics. All chemical materials were used as received without further purification. HPLC grade water was used in all the experiments.

### Preparation of Zn(DDTC) solution

Typically, 2 mmol each of Na(DDTC) and ZnCl_2_ were dissolved in 20 mL of ultrapure water, respectively. The Zn(DDTC) precursor solution was prepared by mixing Na(DDTC) and ZnCl_2_ solution in a 100 mL beaker, then stirred for 2 h to obtain the milky solution. It was then dried in a vacuum drying oven at 60 °C. Finally, 1 mmol of Zn(DDTC) was added to a flask with 10 mL of OAm, and sonicated for 20 min under N_2_ flow.

### Preparation of sulfur solution

The sulfur solution was prepared by reducing 1 mmol of sulfur powder with 5 mL of OAm in a flask. After degassing at room temperature for 10 min, the solution was heated to 100 °C under N_2_ flow, and the temperature was maintained for 10 min. The resulting sulfur solution was then cooled to room temperature for further application.

### Synthesis of CdS/ZnS core/shell QDs

Synthesis of CdS/ZnS was based on a procedure reported previously [37]. The Cd solution was prepared by reducing 0.4 mol of CdO with 1 mL of OA and 15 mL of ODE in a 100 mL three-necked flask at room temperature. The mixture was heated to 260 °C, maintained at 260 °C and then stirred for 10 min under N_2_ flow. The flask was sealed and subsequently 2 mL of sulfur solution was injected to the mixture and the temperature was maintained at 240 °C for 5 min, after that the mixture was cooled to 50 °C. Then 0.5 mL of Zn(DDTC) solution was added to the mixture and the mixture was heated to 180 °C, maintained at 180 °C for 20 min. Finally, the mixture was cooled to room temperature, and separated by the addition of 20 mL of ethanol and by two cycles of centrifugation.

### Preparation of water dispersible micelle-encapsulated QDs

300 μL of CdS/ZnS stock solution in chloroform (2 mg/mL) was mixed with 600 μL of F127 solution (10 mg/mL in chloroform) by gentle stirring for 5 min. The mixture was then evaporated and dried using a roto-evaporator. The residue film was heated to 70–80 °C, and 0.5 mL of water was added to obtain an optically clear suspension. The micelle-encapsulated QDs, F127-CdS/ZnS, were purified by centrifugation and then redispersed in water for further use. F127 is known to stabilize nanospheres and prolong their circulation time in vivo. In this experiment, F127 was used to modify the surface of the CdS/ZnS QDs

### Preparation of in vitro cell imaging

Panc-1 cells were maintained in Dulbecco’s modified Eagle’s medium (DMEM, Sigma-Aldrich) supplemented with 10% fetal bovine serum (FBS, Hyclone) and cultured at 37 °C in a humidified atmosphere with 5% CO_2_. For cell imaging, cells were treated with F127-CdS/ZnS micelles formulations for 2 h. The cells were then washed with PBS for three times and imaged using a Leica confocal microscopy system (TCS SP2). For the cell viability assays, about 5000 cells were dispensed into each well of a 96-well flat-bottom Microtiter plate and cultured overnight. Eight sets were treated with different concentrations of QDs formulations and one set treated with PBS buffer was regarded as the non-treated control. The cells were subsequently incubated for 48 h before the assay. Assays were performed in triplicate and the results were averaged.
